# Single‐cell transcriptome sequencing reveals SPP1‐CD44‐mediated macrophage–tumor cell interactions drive chemoresistance in TNBC

**DOI:** 10.1111/jcmm.18525

**Published:** 2024-07-09

**Authors:** Fuzhong Liu, Junfeng Zhang, Xiaowei Gu, Qiuyang Guo, Wenjia Guo

**Affiliations:** ^1^ Xinjiang Medical University Affiliated Cancer Hospital Urumqi China; ^2^ Anhui Medical University Hefei China; ^3^ Xinjiang Key Laboratory of Translational Biomedical Engineering Urumqi China

**Keywords:** cell communication, chemotherapy resistance, signalling pathway, single‐cell RNA sequencing, triple‐negative breast cancer

## Abstract

Triple‐negative breast cancer (TNBC) is often considered one of the most aggressive subtypes of breast cancer, characterized by a high recurrence rate and low overall survival (OS). It is notorious for posing challenges related to drug resistance. While there has been progress in TNBC research, the mechanisms underlying chemotherapy resistance in TNBC remain largely elusive. We collect single‐cell RNA sequencing (scRNA‐seq) data from five TNBC patients susceptible to chemotherapy and five resistant cases. Comprehensive analyses involving copy number variation (CNV), pseudotime trajectory, cell–cell interactions, pseudospace analysis, as well as transcription factor and functional enrichment are conducted specifically on macrophages and malignant cells. Furthermore, we performed validation experiments on clinical samples using multiplex immunofluorescence. We identified a subset of SPP1^+^ macrophages that secrete SPP1 signals interacting with CD44 on malignant cell surfaces, potentially activating the PDE3B pathway within malignant cells via the integrin pathway, leading to chemotherapy resistance. The abnormally enhanced SPP1 signal between macrophages and malignant cells may serve as a factor promoting chemotherapy resistance in TNBC patients. Therefore, SPP1^+^ macrophages could potentially serve as a therapeutic target to reduce chemotherapy resistance.

## 1 INTRODUCTION

Triple‐negative breast cancer (TNBC) constitutes 15%–20% of breast cancer incidence, predominantly presenting as invasive ductal carcinoma and frequently associated with a dismal prognosis.^1^ Due to the lack of expression of oestrogen receptors, progesterone receptors and human epidermal growth factor receptor 2 (HER2) in TNBC cells, they are insensitive to endocrine therapy and HER2‐targeted treatment. Currently, the standard treatment for the majority of TNBC patients involves neoadjuvant chemotherapy, including agents such as paclitaxel.^2^ Although neoadjuvant chemotherapy is effective for some TNBC patients, approximately 50% of them develop drug resistance, resulting in reduced survival rates.^3^ This resistance may stem from various factors, including tumour cell heterogeneity, alterations in DNA repair mechanisms, the presence of tumour stem cells and the influence of the tumour microenvironment.^4^

Presently, the study of drug resistance often encompasses various aspects, including Bulk‐RNA, metabolomics and proteomics, such as YTHDF1 facilitates S‐phase entry, DNA replication and DNA damage repair,^5^ senescent neutrophils‐derived exosomal piRNA‐17560 enhances the expression of fat mass and obesity‐associated protein (FTO),^6^ expression of a SUMOylation‐deficient mutant MORC2 or administration of SUMO inhibitor,^7^ reduction of miR‐1275,^8^ GATA3 promotes cell viability by decreasing ferroptosis‐related gene CYB5R2 expression.^9^ However, these conventional methods fail to accurately capture individual differences between cells and struggle to effectively detect and analyse crucial cellular subpopulations. scRNA‐seq technology enables the presentation of the expression profile of all genes in the entire genome at the single‐cell level. This assists in identifying and characterizing specific cell subgroups with distinct biological effects and supports the inference of intercellular communication.^10^ Therefore, we will conduct an in‐depth analysis based on scRNA‐seq data.

The tumour microenvironment (TME) is comprised of tumour cells, tumour stromal cells, endothelial cells, immune cells and the non‐cellular components of the extracellular matrix.^11^ Tumour cells, as the central component of the TME, intricately regulate the functions of both cellular and non‐cellular components through complex signalling networks, manipulating non‐malignant cells for their benefit. The repercussions of this crosstalk are reflected in the insufficient response of tumours to treatment, potentially leading to multidrug resistance (MDR).^12^ The source of intercellular communication includes a complex network composed of cytokines, chemokines, growth factors, inflammatory mediators and matrix remodelling enzymes. However, other intriguing mechanisms of interaction are emerging.^13^ Research has shown that features associated with ferroptosis and pyroptosis are closely linked to chemotherapy efficacy.^14^ Mediating the interaction between macrophages and tumour cells can induce chemotherapy resistance in TNBC,^15^ disrupting or interfering with malignant signal transduction in intercellular communication is a strategy to address chemotherapy resistance.^16^ Considering the current state of research and the crucial role of intercellular communication signals in tumours, our study focuses on the role of cell signalling pathways in chemotherapy resistance in triple‐negative breast cancer.

In this study, we analysed scRNA‐seq data from 5 TNBC chemotherapy‐susceptible samples and 5 TNBC chemotherapy‐resistant samples. Intercellular communication analysis revealed an aberrantly active SPP1 signal in the resistant group, and transcription factor analysis identified CEBPB as the upstream regulator of SPP1. Additionally, we identified CD44^+^ malignant cell clusters, which, upon receiving the SPP1 signal, may activate the intracellular PDE3B pathway through FYN‐mediated integrin signalling, leading to chemotherapy resistance. The flowchart and graphical abstract of the research in this paper are shown in Figures S1 and S2.

## 2 MATERIALS AND METHODS

### 2.1 Data acquisition

The scRNA‐seq data utilized in this study were sourced from the GEO database (GSE169246). We obtained scRNA‐seq data from 10 patients diagnosed with TNBC who underwent paclitaxel chemotherapy. Among these 10 patients, we classified patients who exhibited a partial response (PR) according to RECIST 1.1 criteria as sensitive, while those showing stable disease (SD) and progressive disease (PD) were defined as the resistant group.^17^ The outcome revealed five patients in the sensitive group and five patients in the resistant group. Detailed clinical information for all 10 patients can be found in Table S1.

The TNBC samples from both chemosensitive and chemoresistant cases were obtained from the Affiliated Tumor Hospital of Xinjiang Medical University. All six enrolled patients underwent neoadjuvant chemotherapy followed by surgical treatment. This study was approved by the Ethics Committee of the Affiliated Tumor Hospital of Xinjiang Medical University, and informed consent was obtained from all patients. Clinical information of the TNBC patients used in this study is provided in Table S2.

### 2.2 Clustering dimensionality reduction of scRNA‐seq data

We utilized the “Seurat” R package to process the single‐cell data.^18^ For quality control, we excluded cells expressing fewer than 50 genes or with fewer than 300 expressed genes. Subsequently, cells with 200–2500 RNA features were retained. We employed the NormalizeData function in Seurat to obtain normalised counts. Specifically, the global scaling normalisation method “LogNormalize” normalized the gene expression measurements of each cell by multiplying the total expression by a scaling factor (default is 10,000). We then used the FindVariableFeatures function to identify the majority of variable genes by setting nfeatures to 2000. The ScaleData function was applied to transform all genes, ensuring that the mean expression of each gene across all cells was 0 and the variance was 1. Next, principal component analysis (PCA) was performed using the first 20 principal components (PCs), and clustering was executed using the FindClusters function with a resolution of 0.8. Finally, the RunUMAP function was employed to perform uniform manifold approximation and projection (UMAP) analysis, followed by annotation using classical marker genes for different cell types.

### 2.3 Cell–Cell communication analysis

We utilized the R software package CellChat (version 1.6.1) (https://github.com/sqjin/CellChat) to investigate intercellular communication and identify signalling molecules involved in cell‐to‐cell interactions at the single‐cell level.^19^ Initially, the identifyOverExpressedGenes function was employed to detect overexpressed ligands or receptors, followed by the computeCommunProbPathway function to infer communication probabilities. Finally, the netAnalysis_computeCentrality function was utilized to aggregate the communication network. Furthermore, to identify key contributors in the cell–cell communication network, we calculated the centrality scores for each component and presented the results in a heatmap format.

### 2.4 Pseudospatial analysis

We utilized the CSOmap algorithms (https://github.com/lijxug/CSOmapR) to investigate the three‐dimensional pseudospace of distinct cell types.^20^ Initially, the runExactTSNE_R function was employed to compute spatial information for each cell, followed by density value calculations using the getDensity3D function. Finally, we utilized the plot3D function to visualize the spatial distribution of macrophages and malignant cells.

### 2.5 Single‐cell copy number variation analysis

To identify malignant cells within the epithelial population, we assessed copy number variations (CNVs) in each cell across different chromosomal regions using the R package InferCNV.^21^ Using B and T cells as immune cell references, we calculated CNV levels within the epithelial cell clusters. We employed the CreateInfercnvObject function with a cutoff parameter set to 0.1, followed by calculation of CNV values for each cell using the infercnv::run function. Finally, we compared the differences in CNV values between different clusters using boxplots.

### 2.6 Single‐cell trajectory analysis

In this study, we utilized the R package Monocle (version 2.28.0) (http://cole‐trapnell‐lab.github.io/monocle‐release/docs/) for trajectory analysis of cell states to uncover the evolution of cell states.^22^ Initially, the newCellDataSet function in Monocle was employed to convert the Seurat object into a Monocle object. Subsequently, setOrderingFilter and estimateSizeFactors functions were used to construct the developmental trajectory of cells, alongside the reduceDimension function for dimensionality reduction. Finally, the orderCells function was employed to arrange cells along the trajectory. All functionalities were set to default settings.

### 2.7 CytoTRACE analysis

We employed the R package CytoTRACE (version 0.3.3) to predict the relative differentiation status of cells.^23^ Initially, the Idents function was utilized to obtain labels for each cell, followed by the CytoTRACE function to assess the differentiation potential of distinct single‐cell subpopulations. Finally, we utilized the plotCytoTRACE function to visualize the differentiation status of distinct cell clusters.

### 2.8 Pathway analysis

We performed functional enrichment analysis on the identified central cell types using the R package ReactomeGSA (version 1.14.0) (https://github.com/reactome/ReactomeGSA).^24^ We performed enrichment analysis using the “analyse_sc_clusters” function and extracted pathway scores in different cell clusters using the “pathways” function. Finally, we visualized the results using the plot_gsva_heatmap function.

### 2.9 Single‐cell transcription factor analysis

We employed the R package SCENIC (version 1.3.1) (https://github.com/aertslab/SCENIC) to infer the transcription factor regulatory network.^25^ Initially, the “scenicOptions” variable was constructed using the “initializeScenic” function, followed by computing the co‐expression network using the “runSCENIC” function. Finally, the transcription factors' area under the curve (AUC) values across various cells were obtained using the “getAUC” function. We utilized Cytoscape to depict the regulatory network connecting transcription factors and their target genes.

### 2.10 Immunofluorescence staining

The levels of SPP1 and CD44 proteins were detected through mIF. Sections were deparaffinized, underwent antigen retrieval and were blocked with serum to prevent non‐specific binding. Primary antibodies targeting the genes of interest were applied overnight at 4°C, followed by washing and incubation with fluorescently labelled secondary antibodies for 1–2 h at room temperature. Nuclear staining was performed with appropriate dyes if necessary. Sections were then mounted and visualized using a fluorescence/confocal microscope.

### 2.11 Statistical analysis

This study conducted all statistical analyses using R (version 4.3.1). Gene expression differences were assessed using *t*‐tests and Wilcoxon rank‐sum tests. The Pearson correlation coefficient is employed to assess the relationship between two variables. Statistical significance was represented by two‐tailed *p*‐values, where *p* < 0.05 indicated a statistically significant difference.

## 3 RESULTS

### 3.1 Establishment of the single‐cell landscape in TNBC

**FIGURE 1:**
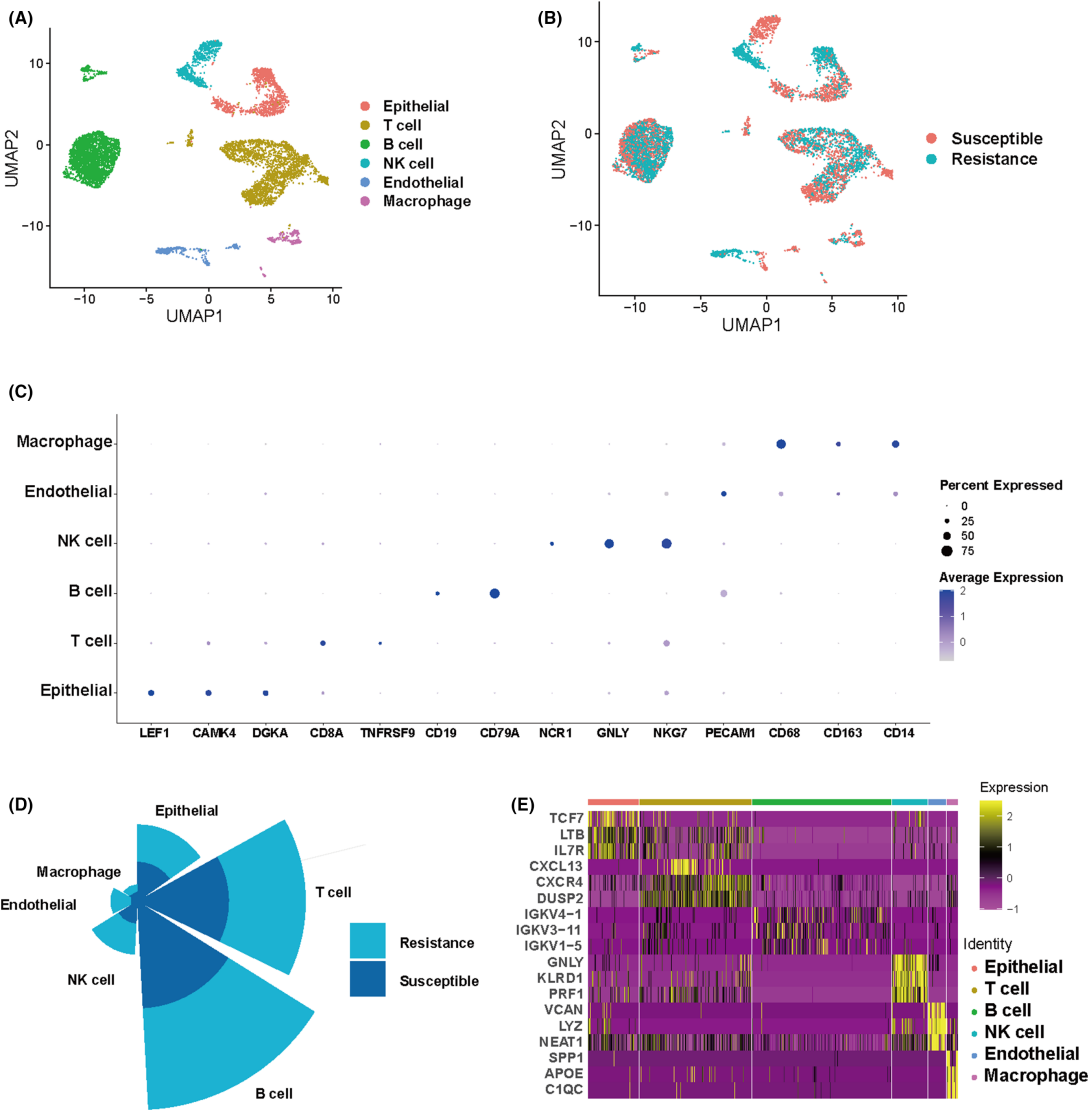
UMAP dimensionality reduction and annotation. (A) UMAP plots delineating six cell types. (B) UMAP plots indicating Susceptible and Resistance. (C) Bubble chart annotating cell types. (D) Quantity proportions of distinct cell types. (E) Heatmap showcasing the expression of marker genes across the six cell types.

To construct the single‐cell landscape of TNBC, we performed a comprehensive analysis of the scRNA‐seq data from 5 chemotherapy‐susceptible and 5 chemotherapy‐resistant patients retrieved from the GEO database (GSE169246). Following stringent quality control (QC), we identified 7585 high‐quality cells and 18,089 genes. Cell type annotation using marker genes^26^ confirmed six distinct cell types: macrophage, endothelial, NK cell, B cell, T cell and epithelial (Figure 1A). Subsequently, cells were segregated into susceptible (*n* = 3863) and resistant (*n* = 3722) groups, with roughly equal cell numbers in both groups (Figure 1B). Bubble plots illustrated the expression profiles of marker genes across different cell clusters: macrophage (CD68, CD163 and CD14), endothelial (PECAM1), NK cell (NCR1, GNLY and NKG7), B cell (CD19, CD79A), T cell (CD8A, TNFRSF9) and epithelial (LEF1, CAMK4, DGK4) (Figure 1C). A rose plot intuitively depicted the relative proportions of different cell types, with B cells being the most abundant (*n* = 2876), followed by T cells (*n* = 2317), epithelial cells (*n* = 1058), NK cells (*n* = 733), endothelial cells (*n* = 370) and macrophages (*n* = 231) (Figure 1D). Following this, we identified marker genes for each cell type and represented their expression levels using heatmaps (Figure 1E).

### 3.2 Identification of malignant cell clusters

**FIGURE 2:**
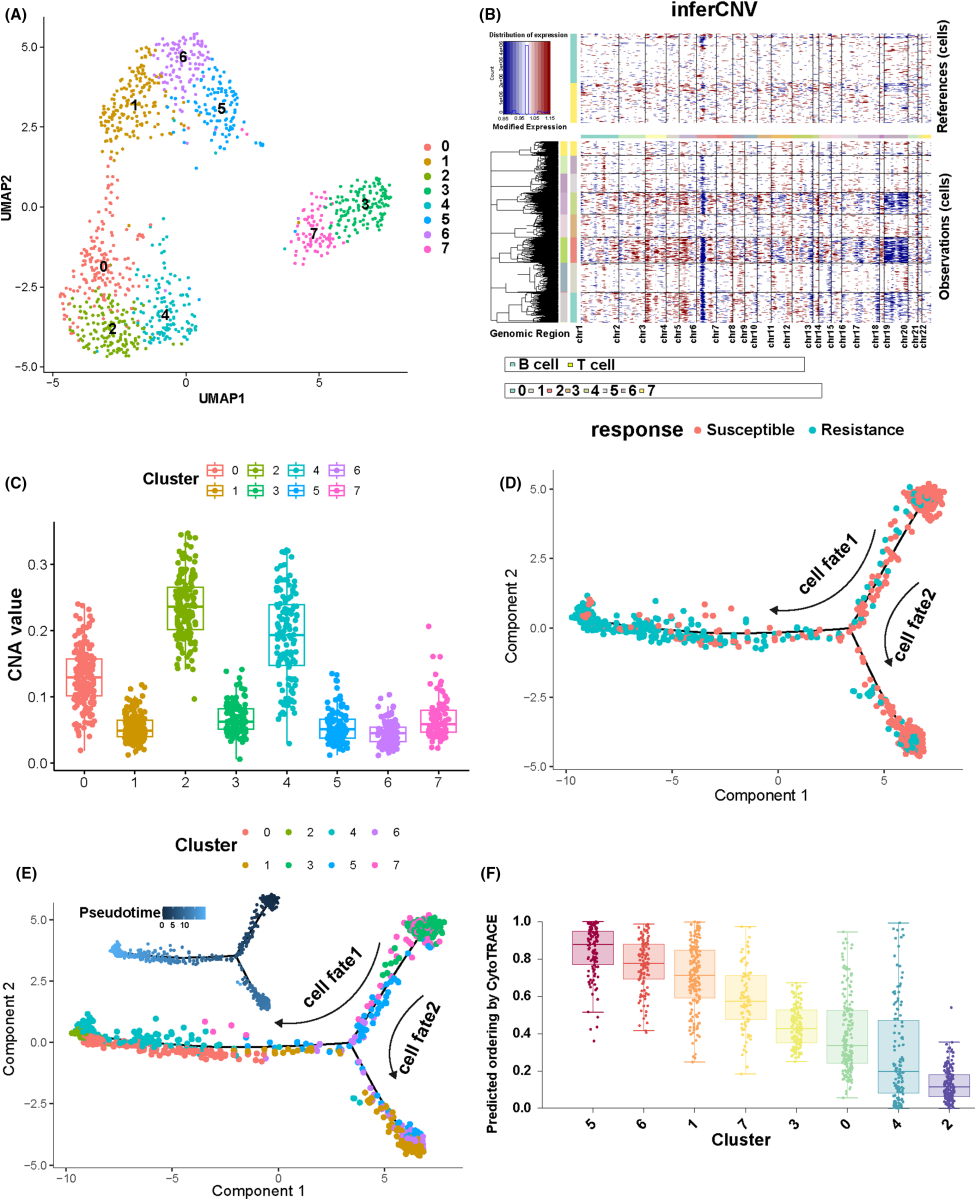
Identification of malignant cell clusters. (A) UMAP plots depicting Epithelial cells. (B) Heatmap displaying CNV analysis results. Red denotes chromosomal CNV amplifications, blue indicates CNV deletions and the intensity of colour reflects the magnitude of CNV variation. (C) Boxplot showing differences in CNV values among different cell subgroups. (D) Distribution of sensitive and resistant cells along the trajectory. (E) Pseudotime analysis showcasing cell progression, where lighter colours indicate proximity to the final cell trajectory stage. (F) Analysis of epithelial cell differentiation status using CytoTRACE.

After extracting 1058 epithelial cells, we performed UMP dimensionality reduction analysis and reclustered the epithelial cells into 8 clusters (Figure 2A). Breast cancer cells originate from epithelial cells, and to investigate which clusters represent malignant cells, we conducted CNV analysis on these 8 clusters. High levels of CNVs are closely associated with cancer development, allowing the identification of potentially malignant cells based on their CNV.^27^ Utilizing a reference set comprising 5193 cells, including T cells and B cells, we noted a substantial increase in CNV levels in clusters 0, 2 and 4 compared to other clusters. Therefore, clusters 0, 2 and 4 were annotated as clusters of malignant cells (Figure 2B,C). Subsequently, we constructed the differentiation trajectory of epithelial cell clusters composed of cellfate1 and cellfate2. Notably, cellfate1 reflected the process of cells transitioning from sensitivity to resistance, while cellfate2 represented the ongoing sensitivity of cells (Figure 2D). Continuing, we investigated the positioning of identified malignant cell clusters along the differentiation trajectory and found a significant enrichment of clusters 0, 2 and 4 at the endpoint of cell fate 1. (Figure 2E). Using the CytoTRACE algorithm, each cell obtained a score ranging from 0 to 1.^28^ After conducting CytoTRACE analysis on the eight clusters of epithelial cells, the results demonstrated that clusters 0,2 and 4 were at the ends of the trajectory (Figure 2F). These findings suggest that clusters 0, 2 and 4 represent malignant cells associated with breast cancer, exhibiting distinct resistance characteristics during cellular differentiation, with cluster 2 potentially representing the subset of malignant cells with the highest degree of differentiation.

### 3.3 Identifying transcription factors in SPP1 ^+^ macrophage clusters

**FIGURE 3:**
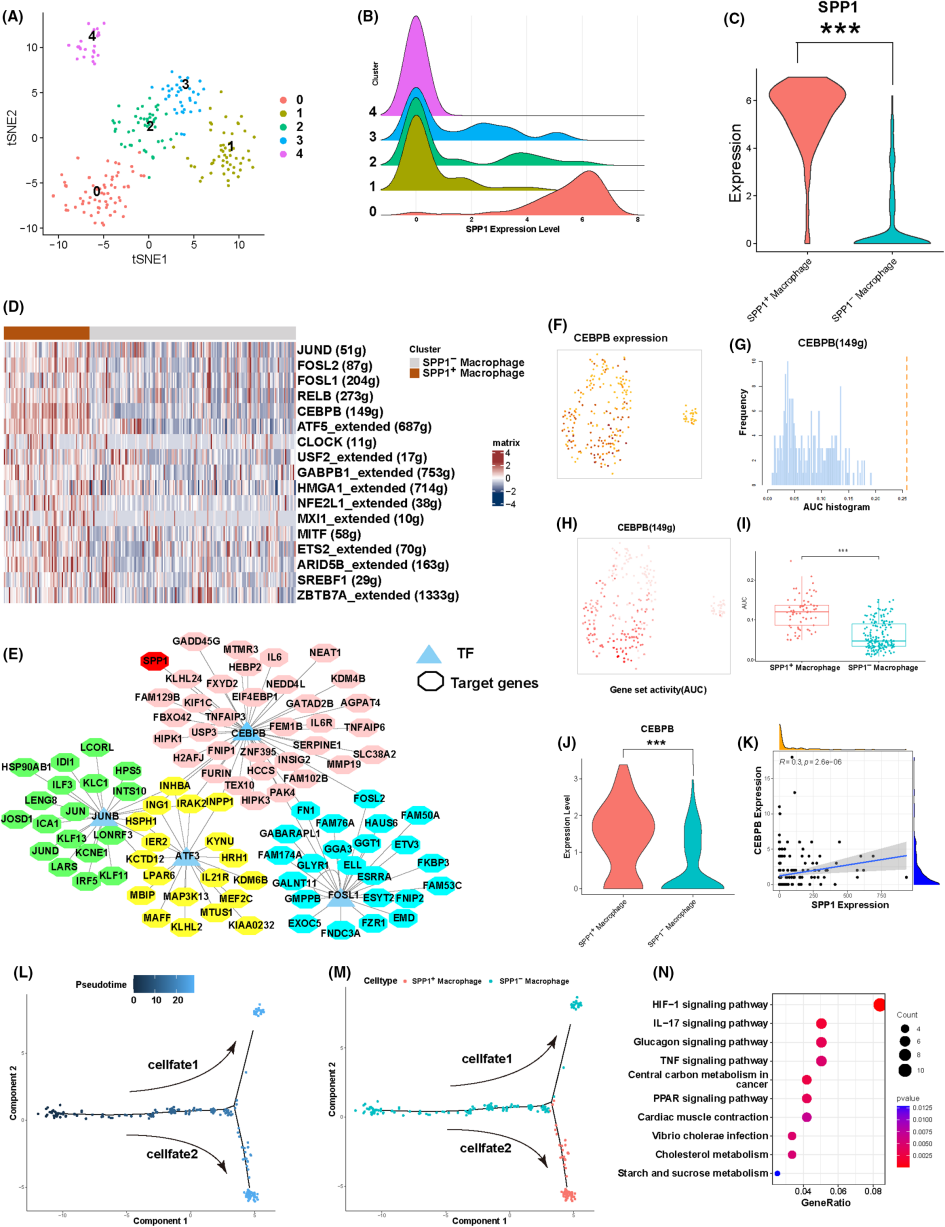
Transcription factor analysis of macrophages. (A) UMAP plots of Macrophage. (B) Mountain plot displaying the differential expression of SPP1 in macrophage subtypes. (C) Expression disparity of SPP1 in SPP1^+^ and SPP1^−^ clusters. (D) Heatmap showing the enrichment of transcription factor activity among distinct cell clusters. (E) Regulatory network of transcription factors identifying the pivotal transcription factor CEBPB. (F–H) Assessment of CEBPB activity using AUCell. (I) Boxplot depicting the divergence in AUC values of CEBPB between the two clusters. (J) Violin plot showcasing the expression variance of CEBPB across the two clusters. (K) Correlation analysis between CEBPB and SPP1. (L) Potential trajectory of macrophages identified two distinct cell fates. (M) Distribution of different macrophage clusters along the trajectory. (N) KEGG enrichment analysis of the marker genes of SPP1^+^ macrophages. *p*‐Values are denoted as: *p < 0.05; **p < 0.01; ***p < 0.001.

We performed UMP dimensionality reduction analysis on extracted macrophages, clustering them into 5 distinct clusters (Figure 3A). Research indicates that SPP1 influences the polarisation state of macrophages,^29^ and the interaction between SPP1 and macrophages may play a crucial role in the chemotherapy response of tumours.^30^ Hence, we proceeded with the comparative analysis of SPP1 expression across the five clusters. The results revealed a significantly higher expression level of SPP1 in cluster 0 compared to the other clusters (Figure 3B). Based on this, we defined cluster 0 as SPP1^+^ macrophage cluster, while the remaining clusters were defined as SPP1^−^ macrophage clusters. To investigate the regulatory mechanisms of SPP1, we employed the SCENIC R package (v1.3.1) for transcription factors (TFs) identification in macrophages.^31^ We identified a total of 142 transcription factors, with particular focus on 17 highly transcriptionally active factors significantly enriched in the SPP1^+^ macrophage cluster (Figure 3D). Further analysis of the gene regulatory network revealed CEBPB as a key regulatory factor for SPP1 (Figure 3E). Subsequently, we quantified the activity of the transcription factor CEBPB using AUCell and found that the area under the curve (AUC) of CEBPB was significantly higher in SPP1^+^ macrophage clusters compared to SPP1^−^ macrophage clusters (Figure 3F–I). Furthermore, compared to SPP1^−^ macrophage clusters, the expression level of CEBPB was higher in SPP1^+^ macrophages (Figure 3J). Finally, correlation analysis demonstrated a moderate correlation between CEBPB and SPP1 expression levels (*r* = 0.3, *p* < 0.001) (Figure 3K). After trajectory analysis of macrophages, we observed distinct distribution patterns between two clusters formed by cellfate1 and cellfate2. SPP1^+^ macrophages were predominantly enriched in cellfate2, whereas SPP1^−^ macrophages were mainly enriched in cellfate1 (Figure 3L,M). KEGG analysis of marker genes of SPP1^+^ macrophages revealed enrichment of pathways such as HIF‐1^32^ and IL‐17,^33^ which have been associated with chemoresistance, according to relevant studies (Figure 3N). In summary, these analyses emphasize the crucial role of SPP1 in macrophages, particularly through regulation by CEBPB.

### 3.4 Characterization of SPP1‐CD44 by the ligand receptor

**FIGURE 4:**
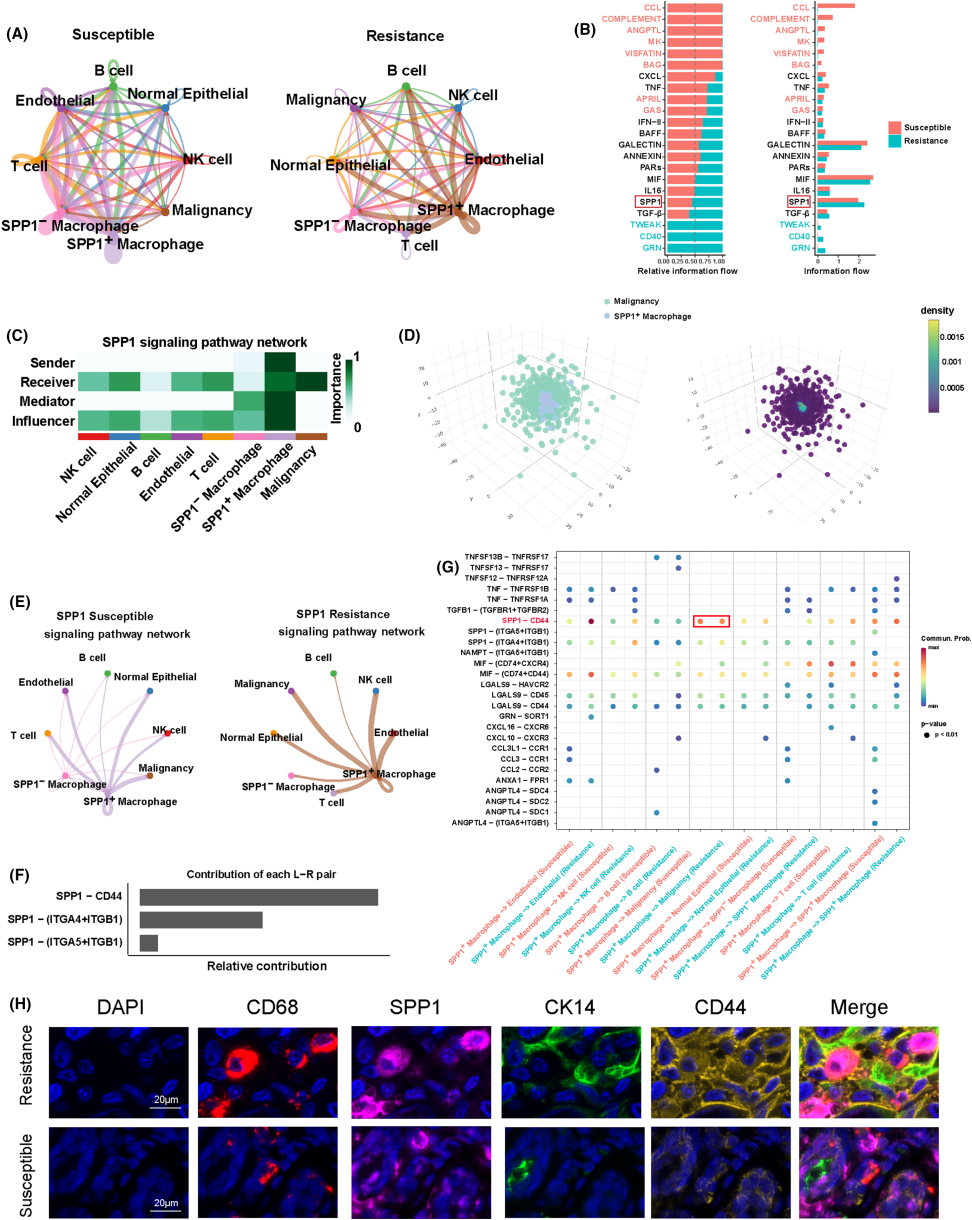
Analysis of cellular communication for 8 cell types. (A) Circular plot depicting interactions among eight cell types. (B) Bar graph displaying differences in information flow, where dark red represents enrichment of signals containing susceptible, and cyan represents signals enriched in resistance. (C) Heatmap illustrating the network centrality scores of the SPP1 signalling pathway. (D) Virtual spatial positioning and density between SPP1^+^macrophage and CD44^+^ Malignancy. (E) Network plot illustrating cell–cell interactions within the SPP1 signalling pathway. (F) Bar chart depicting the ligand‐receptor pairs mediating SPP1 signal transduction. (G) Bubble plot displaying the strength of intercellular signalling for different ligand‐receptor pairs. (H) Multiplex immunofluorescence was used to detect the expression of CD68, SPP1, CK14 and CD44 in chemoresistant and chemosensitive samples. Scale bars: 100 μm.

To identify active signals in the resistant group, we employed the CellChat R package to build a cell–cell communication network involving eight cell types, including SPP1^+^ macrophages and malignant cell clusters (Figure 4A). The results indicate that the intensities of the SPP1 and TGF‐β signals are higher in the resistant group compared to the sensitive group, with SPP1 showing a significantly higher signal intensity than TGF‐β. Therefore, we chose to conduct further analysis on the SPP1 signal (Figure 4B). Furthermore, through the calculation of network centrality measures for each cell group, we determined that SPP1^+^ macrophages serve as the principal signal senders, while malignant cells act as the receivers in this cell communication network (Figure 4C). Using the CSOmap algorithm based on the cellular expression profiles of SPP1^+^ macrophages and malignant cells, we explored the three‐dimensional pseudospace, indicating a close primary connection structure and mutual closure between SPP1^+^ macrophages and malignant cells in pseudospace (Figure 4D). Additionally, the cell communication network in the SPP1 signalling pathway revealed a high level of signal intensity between SPP1^+^ macrophage clusters and malignant cell clusters (Figure 4E). In summary, these findings suggest a crucial role for the SPP1 signalling pathway in the resistance mechanism, potentially influencing the chemotherapeutic resistance of tumour cells through cell–cell communication between SPP1^+^ macrophages and malignant cells.

Within the SPP1 signalling pathway, three crucial ligand–receptor pairs were identified: SPP1‐CD44, SPP1‐(ITGA4 + ITGB1) and SPP1‐(ITGA5 + ITGB1) (Figure 4F). To investigate which ligand‐receptor pair plays a role in mediating the resistant SPP1 signal, we separately calculated the communication probabilities of different ligand‐receptor pairs between susceptible and resistant groups. The results indicated a markedly higher communication probability for the ligand‐receptor pair SPP1‐CD44 compared to the other two pairs (Figure 4G). As this ligand‐receptor pair is within the SPP1 signalling pathway, its signal sender and receiver align with the SPP1 signalling pathway, represented by SPP1^+^ macrophages and malignant cells, respectively. Finally, in clinical samples of TNBC, we observed that the fluorescence intensity of SPP1 and CD44 in chemoresistant samples was significantly higher than in chemosensitive samples. Moreover, SPP1‐expressing macrophages (CD68^+^) and CD44‐expressing malignant cells (CK14^+^) were closer together in chemotherapy‐resistant samples, suggesting a potential interaction between these two cells (Figure 4H). These findings suggest that the secretion of SPP1 by SPP1^+^ macrophages, when bound to CD44 on the surface of malignant cells, influences the chemotherapeutic resistance of tumour cells.

### 3.5 Recognition of CD44 ^+^ malignant cell cluster transcription factors

**FIGURE 5:**
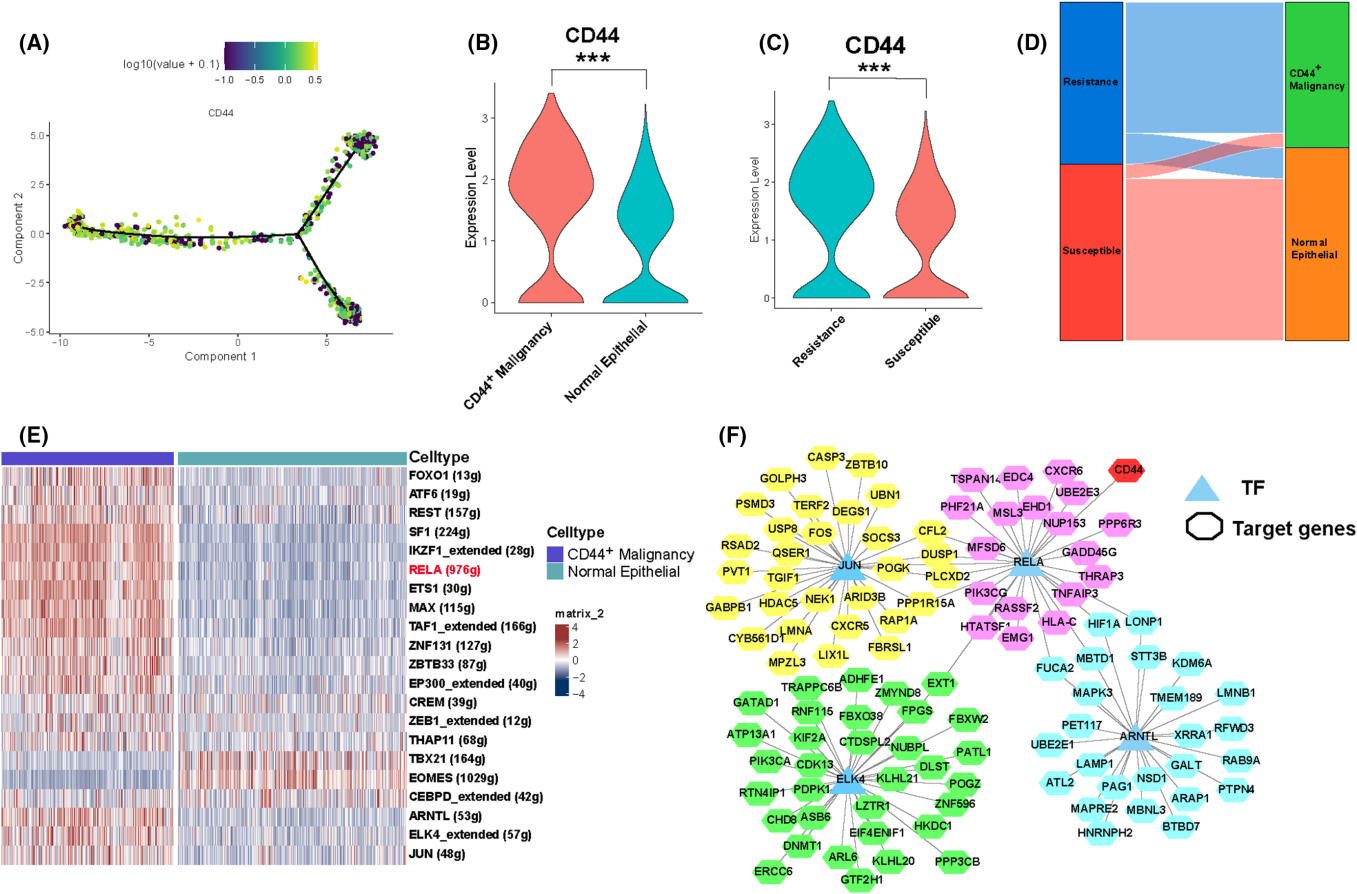
Transcription factor analysis of malignant cell. (A) Pseudotime trajectory exhibits the dynamic expression changes of CD44. (B) A violin plot was employed to compare the expression difference of CD44 between CD44^+^malignant cells and normal epithelial cells. (C) A violin plot was used to compare the expression difference of CD44 between susceptible and resistant groups within epithelial cells. (D) Sankey diagram displays the association between the two categorized groups. (E) Heatmap illustrates the enrichment of transcription factor activity in the CD44^+^malignant. (F) Transcription factor regulatory network identifies the key transcription factor RELA. *p* Values were denoted as: *p < 0.05; **p < 0.01; ***p < 0.001.

In response to the previously identified CD44 receptor in cell communication analysis, we further investigated its expression in malignant cells. The results revealed that in cellfate1, the transition from susceptible to resistant cells, the expression of CD44 gradually increased (Figure 5A). Moreover, the expression of CD44 in malignant cells was significantly higher than in normal epithelial cells (Figure 5B). Therefore, we defined clusters 0, 2 and 4 of malignant cells as CD44^+^ malignant cell clusters. Interestingly, in epithelial cells, the expression of CD44 in the resistant group was also significantly higher than in the susceptible group (Figure 5C). Comparison of associations between different phenotypes using a stream plot indicated that the majority of CD44^+^ malignant cell clusters belonged to the resistant cells, with only a small fraction showing sensitivity (Figure 5D). These findings suggest that CD44 may influence the chemotherapeutic resistance of tumours. To gain a deeper understanding of the regulatory mechanisms of the CD44 signalling pathway, we utilized SCENIC clustering technology for the identification of transcription factors. Among them, 21 transcription factors, including FOXO1 and ATF6, exhibited significant differences in the AUC values between CD44^+^ malignant cell clusters and normal epithelial cell clusters (Figure 5E).^34^ Further regulatory network analysis revealed that RELA's target genes included the key receptor CD44 of the SPP1 pathway (Figure 5F). These findings suggest that the transcription factor RELA influences the drug resistance of malignant cells by regulating CD44.

### 3.6 SPP1 signalling pathway triggers intracellular signalling in target cells

**FIGURE 6:**
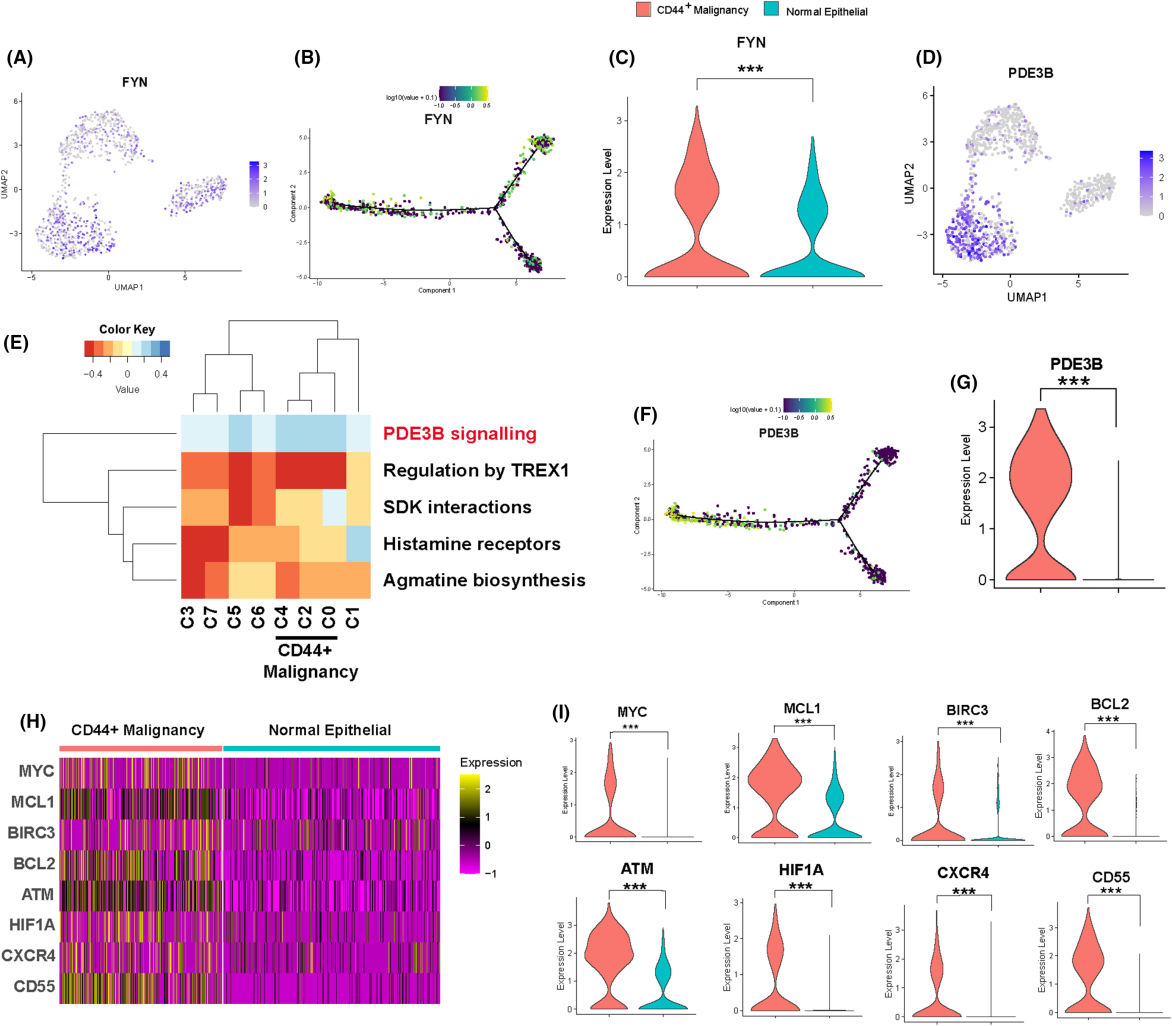
The SPP1 signalling pathway triggers intracellular signal transduction in malignant cells. (A) UMAP plots demonstrate the expression of FYN across cell clusters. (B) Pseudotime trajectory exhibits the dynamic expression changes of FYN. (C) Violin plots compare the expression differences of FYN between CD44^+^malignant cells and normal epithelial cells. (D) UMAP plots display the expression of FYN across cell clusters. (E) Heatmaps illustrate the activated signalling pathways in different cell clusters. (F) Pseudotime trajectory depicts the dynamic expression changes of PDE3B. (G) Violin plots compare the expression differences of PDE3B between malignant cells and normal epithelial cells. (H) Heatmaps display the enrichment of marker genes associated with biological behaviours such as tumour occurrence, proliferation in CD44^+^malignant cells and normal epithelial cells. (I) Violin plots demonstrate the gene expression differences of markers associated with tumour biology in CD44^+^malignant cells and normal epithelial cells. *p*‐Values are indicated as: *p < 0.05; **p < 0.01; ***p < 0.001.

To investigate how the SPP1 signal transduces to malignant cells, leading to chemotherapy resistance, we conducted further analysis on the CD44^+^ malignant cell cluster. Integrins are membrane proteins that interact with extracellular matrix molecules, participating in the connection between cells and the external environment.^35^ When integrins interact with specific extracellular activating factors (such as FAK, SRC family kinases and ILK), they can aggregate and trigger downstream signalling pathways, modulating various cellular functions like migration, proliferation and apoptosis.^36^ We observed that FYN,^37^ a member of the SRC kinase family, was significantly overexpressed in the CD44^+^ malignant cell cluster compared to normal epithelial cells (Figure 6A–C). Subsequently, we utilized the R package ReactomeGSA to analyse downstream signals activated by integrin, unveiling a notable activation of the PDE3B signalling pathway was observed in the CD44^+^malignant cell cluster (Figure 6E). Consistent with CD44 and FYN, a key member of the PDE3B signalling pathway, PDE3B, exhibited significantly higher expression in the CD44^+^ malignant cell cluster than in normal epithelial cells (Figure 6F,G). We subsequently explored marker genes linked to the biological behaviour of cancer cells, identifying the enrichment of eight marker genes in CD44^+^ malignant cell clusters (Figure 6H,I). These marker genes play diverse roles in cancer, such as promoting growth and proliferation (MYC, HIF1A, ATM), inhibiting apoptosis (MCL1, BIRC3, BCL2) and facilitating invasion (CXCR4, CD55). These results suggest that the SPP1 signal secreted by macrophages, upon binding to the CD44 receptor on the surface of CD44^+^ malignant cells, may induce chemotherapy resistance by activating intracellular signals such as FYN and PDE3B.

## 4 DISCUSSION

In our study, analysis of scRNA‐seq data from both susceptible and resistant groups of breast cancer chemotherapy revealed an abnormally active signal, SPP1, secreted by macrophages. This signal forms a complex with the receptor CD44 on cancer cell surfaces, activating the PDE3B signalling pathway through the integrin enzyme pathway, consequently leading to the resistance of cancer cells to chemotherapy drugs.

Studies have demonstrated CEBPB's involvement in chemotherapy resistance among TNBC patients, where LINC00160 modulates chemoresistance by recruiting CEBPB to the TFF3 promoter, augmenting TFF3 expression.^38^ Consistent with our findings, we reveal CEBPB's impact on breast cancer chemoresistance through the SPP1 signalling pathway. In the realm of immunity, inhibiting glycolysis in breast cancer patients via the CEBPB pathway impedes G‐CSF and GM‐CSF production, reducing myeloid‐derived suppressor cells (MDSCs) and enhancing anti‐tumour immunity.^39^ Additionally, CEBPB regulates breast cancer cell migration and invasion through diverse pathways, including THBS2 suppression, the PAK4–CEBPB–CLDN4 axis and the cAMP/AMPK/CEBPB axis.^40^ PDPN‐positive CAFs could represent a novel therapeutic target for overcoming resistance in HER2‐positive breast cancer.^41^

Identifying potential therapeutic targets for triple‐negative breast cancer is of paramount importance.^42^ Through cell communication analysis, we identified the SPP1 signal, known to predict breast cancer recurrence post‐tamoxifen treatment. Elevated SPP1 levels correlate with adverse breast cancer prognosis, aligning with our prognostic research direction.^43^ Mechanistically, downregulation of miR‐944^44^ and miR‐127^45^ promotes high SPP1 expression, fostering cancer progression via direct pathway induction and interaction with growth factor receptor pathways, activating genes conducive to cancer advancement.CD44, a non‐kinase transmembrane glycoprotein, acts as a receptor for SPP1 in this study.^46^ This pairing has also been observed in pancreatic,^47^ liver cancers,^48^ and clear cell renal cell carcinoma.^49^ Studies indicate CD44 as a shared marker for cancer stem cells in breast cancer, with high CD44 expression correlating with adverse BC prognosis,^50^ consistent with our findings. In doxorubicin resistance mechanisms, CD44 plays a role.^51^ Downstream, CD44 can activate Ras‐ERKs and PI3K‐AKT pathways,^52^ influencing cancer cell proliferation and motility. Additionally, recent studies have identified a highly potent small‐molecule antagonist of exportin‐1, which selectively eliminates CD44^+^CD24^−^ enriched breast cancer stem‐like cells.^53^ FYN, a tyrosine kinase, participates in the intracellular signalling cascade, facilitating the transportation of various cell surface receptors.^54^ Studies indicate FYN's involvement in activating downstream pathways like RafERK/MAPK,^55^ PI3K/Akt, abnormal NF‐κB signalling,^56^ and JAK/STAT pathways. Moreover, FYN contributes to chemotherapy resistance through adhesive‐mediated mechanisms.

While our study results contribute new insights into the mechanisms of chemotherapy resistance in breast cancer, it is important to acknowledge certain potential limitations. Firstly, our analysis is based on data from public databases with a limited sample size, which may introduce some degree of selection bias. Furthermore, although we conducted preliminary validation of the functionality of CD44^+^ tumour cells, the roles of the key signal SPP1 and its upstream transcriptional regulatory mechanisms, as well as downstream signalling transduction mechanisms, lack validation through in vivo and in vitro experiments. Lastly, single‐cell RNA sequencing data suffers from a lack of inherent cellular spatial information, posing challenges in delivering comprehensive and representative spatial details.

In this study, we delineated the landscape of the entire tumour microenvironment in TNBC using scRNA‐seq, identifying macrophages as crucial mediators of communication with malignant cells. Macrophages constitute a major component of the TME, with distinct subsets exhibiting varied functionalities.^57^ MRC1^+^ macrophages facilitate tumour recurrence following chemotherapy, and tumour biopsy samples from cancer patients who received neoadjuvant therapy had a much larger infiltrate of CD45^+^CD11b^+^CD14^+^ macrophages than those from patients who received only surgery.^58^, ^59^ Therefore, precise characterization of macrophage phenotypes and functions is crucial. We identified a novel subset of macrophages that, through secretion of SPP1, bind to CD44 on tumour cells, activating the PDE3B pathway via the integrin enzyme pathway, thereby inducing chemotherapy resistance in TNBC patients. Our study, akin to prior research, characterised distinct macrophage subpopulations, potentially aiding in the development of precise therapeutic strategies targeting SPP1^+^ macrophages to minimize chemotherapy resistance.

## AUTHOR CONTRIBUTIONS

**Fuzhong Liu:** Data curation (equal); software (equal); writing – original draft (equal). **Junfeng Zhang:** Data curation (equal). **Xiaowei Gu:** Conceptualization (equal); resources (equal). **Qiuyang Guo:** Data curation (equal); software (equal). **Wenjia Guo:** Conceptualization (equal); software (equal); writing – review and editing (equal).

## FUNDING INFORMATION

This study was supported by the Xinjiang Medical University Scientfic Research Innovation Team Project (XYD‐2024C09), Xinjiang Uygur Autonomous Region Natural Science Foundation (2022D01E27), Tianchi Young Talent Doctoral Program (2022TCYCGWJ), Natural Science Foundation of Xinjiang Uygur Autonomous Region (2023D01C124) and The National Natural Science Foundation of China (82260791).

## CONFLICT OF INTEREST STATEMENT

All authors declare no conflict of interest.

## Supporting information


Figure S1.

Figure S2.

Table S1.

Table S2.

## Data Availability

All data are available in a public, open access repository. R and other custom scripts for analysing data are available upon reasonable request.

## References

[1] Borri F , Granaglia A . Pathology of triple negative breast cancer. Semin Cancer Biol. 2021;72:136‐145. doi:10.1016/j.semcancer.2020.06.005 32544511

[2] van den Ende NS , Nguyen AH , Jager A , Kok M , Debets R , van Deurzen CHM . Triple‐negative breast cancer and predictive markers of response to neoadjuvant chemotherapy: a systematic review. Int J Mol Sci. 2023;24:2969. doi:10.3390/ijms24032969 36769287 PMC9918290

[3] Kim C , Gao R , Sei E , et al. Chemoresistance evolution in triple‐negative breast cancer delineated by single‐cell sequencing. Cell. 2018;173:879‐893.e813. doi:10.1016/j.cell.2018.03.041 29681456 PMC6132060

[4] Ferrari P , Scatena C , Ghilli M , Bargagna I , Lorenzini G , Nicolini A . Molecular mechanisms, biomarkers and emerging therapies for chemotherapy resistant TNBC. Int J Mol Sci. 2022;23:1665. doi:10.3390/ijms23031665 35163586 PMC8836182

[5] Sun Y , Dong D , Xia Y , Hao L , Wang W , Zhao C . YTHDF1 promotes breast cancer cell growth, DNA damage repair and chemoresistance. Cell Death Dis. 2022;13:230. doi:10.1038/s41419-022-04672-5 35279688 PMC8918344

[6] Ou B , Liu Y , Gao Z , et al. Senescent neutrophils‐derived exosomal piRNA‐17560 promotes chemoresistance and EMT of breast cancer via FTO‐mediated m6A demethylation. Cell Death Dis. 2022;13:905. doi:10.1038/s41419-022-05317-3 36302751 PMC9613690

[7] Zhang FL , Yang SY , Liao L , et al. Dynamic SUMOylation of MORC2 orchestrates chromatin remodelling and DNA repair in response to DNA damage and drives chemoresistance in breast cancer. Theranostics. 2023;13:973‐990. doi:10.7150/thno.79688 36793866 PMC9925317

[8] Han X , Li M , Xu J , et al. miR‐1275 targets MDK/AKT signaling to inhibit breast cancer chemoresistance by lessening the properties of cancer stem cells. Int J Biol Sci. 2023;19:89‐103. doi:10.7150/ijbs.74227 36594100 PMC9760432

[9] Zhu Z , Shen H , Xu J , et al. GATA3 mediates doxorubicin resistance by inhibiting CYB5R2‐catalyzed iron reduction in breast cancer cells. Drug Resist Updat. 2023;69:100974. doi:10.1016/j.drup.2023.100974 37230023

[10] Bridges K , Miller‐Jensen K . Mapping and validation of scRNA‐seq‐derived cell‐cell communication networks in the tumor microenvironment. Front Immunol. 2022;13:885267. doi:10.3389/fimmu.2022.885267 35572582 PMC9096838

[11] Jahanban‐Esfahlan R , Seidi K , Banimohamad‐Shotorbani B , Jahanban‐Esfahlan A , Yousefi B . Combination of nanotechnology with vascular targeting agents for effective cancer therapy. J Cell Physiol. 2018;233:2982‐2992. doi:10.1002/jcp.26051 28608554

[12] Hanahan D , Coussens LM . Accessories to the crime: functions of cells recruited to the tumor microenvironment. Cancer Cell. 2012;21:309‐322. doi:10.1016/j.ccr.2012.02.022 22439926

[13] Denisenko TV , Budkevich IN , Zhivotovsky B . Cell death‐based treatment of lung adenocarcinoma. Cell Death Dis. 2018;9:117. doi:10.1038/s41419-017-0063-y 29371589 PMC5833343

[14] Zhou L , Wong C , Liu Y , Jiang W , Yang Q . Development and validation of stable ferroptosis‐ and pyroptosis‐related signatures in predicting prognosis and immune status in breast cancer. J Cell Mol Med. 2023;27:3827‐3838. doi:10.1111/jcmm.17958 37849388 PMC10718145

[15] Li H , Yang P , Wang JH , et al. HLF regulates ferroptosis, development and chemoresistance of triple‐negative breast cancer by activating tumor cell‐macrophage crosstalk. J Hematol Oncol. 2022;15:2. doi:10.1186/s13045-021-01223-x 34991659 PMC8740349

[16] Song G , Shi Y , Zhang M , et al. Global immune characterization of HBV/HCV‐related hepatocellular carcinoma identifies macrophage and T‐cell subsets associated with disease progression. Cell Discov. 2020;6:90. doi:10.1038/s41421-020-00214-5 33298893 PMC7721904

[17] Nishino M , Ramaiya NH , Chambers ES , et al. Immune‐related response assessment during PD‐1 inhibitor therapy in advanced non‐small‐cell lung cancer patients. J Immunother Cancer. 2016;4:84. doi:10.1186/s40425-016-0193-2 28018599 PMC5168591

[18] Hao Y , Hao S , Andersen‐Nissen E , et al. Integrated analysis of multimodal single‐cell data. Cell. 2021;184:3573‐3587.e3529. doi:10.1016/j.cell.2021.04.048 34062119 PMC8238499

[19] Jin S , Guerrero‐Juarez CF , Zhang L , et al. Inference and analysis of cell–cell communication using CellChat. Nat Commun. 2021;12:1088. doi:10.1038/s41467-021-21246-9 33597522 PMC7889871

[20] Zhang L , Li Z , Skrzypczynska KM , et al. Single‐cell analyses inform mechanisms of myeloid‐targeted therapies in colon cancer. Cell. 2020;181:442‐459.e429. doi:10.1016/j.cell.2020.03.048 32302573

[21] Jerby‐Arnon L , Shah P , Cuoco MS , et al. A cancer cell program promotes T cell exclusion and resistance to checkpoint blockade. Cell. 2018;175:984‐997.e924. doi:10.1016/j.cell.2018.09.006 30388455 PMC6410377

[22] Qiu X , Mao Q , Tang Y , et al. Reversed graph embedding resolves complex single‐cell trajectories. Nat Methods. 2017;14:979‐982. doi:10.1038/nmeth.4402 28825705 PMC5764547

[23] Gulati GS , Sikandar SS , Wesche DJ , et al. Single‐cell transcriptional diversity is a hallmark of developmental potential. Science. 2020;367:405‐411. doi:10.1126/science.aax0249 31974247 PMC7694873

[24] Griss J , Viteri G , Sidiropoulos K , Nguyen V , Fabregat A , Hermjakob H . ReactomeGSA–efficient multi‐omics comparative pathway analysis. Mol Cell Proteomics. 2020;19:2115‐2125. doi:10.1074/mcp.TIR120.002155 32907876 PMC7710148

[25] Aibar S , González‐Blas CB , Moerman T , et al. SCENIC: single‐cell regulatory network inference and clustering. Nat Methods. 2017;14:1083‐1086. doi:10.1038/nmeth.4463 28991892 PMC5937676

[26] Sun HF , Li LD , Lao IW , et al. Single‐cell RNA sequencing reveals cellular and molecular reprograming landscape of gliomas and lung cancer brain metastases. Clin Transl Med. 2022;12:e1101. doi:10.1002/ctm2.1101 36336787 PMC9637666

[27] Puram SV , Tirosh I , Parikh AS , et al. Single‐cell transcriptomic analysis of primary and metastatic tumor ecosystems in head and neck cancer. Cell. 2017;171:1611‐1624.e1624. doi:10.1016/j.cell.2017.10.044 29198524 PMC5878932

[28] Zhang Z , Wang ZX , Chen YX , et al. Integrated analysis of single‐cell and bulk RNA sequencing data reveals a pan‐cancer stemness signature predicting immunotherapy response. Genome Med. 2022;14:45. doi:10.1186/s13073-022-01050-w 35488273 PMC9052621

[29] Bill R , Wirapati P , Messemaker M , et al. CXCL9:SPP1 macrophage polarity identifies a network of cellular programs that control human cancers. Science. 2023;381:515‐524. doi:10.1126/science.ade2292 37535729 PMC10755760

[30] Matsubara E , Komohara Y , Esumi S , et al. SPP1 derived from macrophages is associated with a worse clinical course and chemo‐resistance in lung adenocarcinoma. Cancers (Basel). 2022;14:4374. doi:10.3390/cancers14184374 36139536 PMC9496817

[31] Van de Sande B , Flerin C , Davie K , et al. A scalable SCENIC workflow for single‐cell gene regulatory network analysis. Nat Protoc. 2020;15:2247‐2276. doi:10.1038/s41596-020-0336-2 32561888

[32] Zhao J , du F , Luo Y , Shen G , Zheng F , Xu B . The emerging role of hypoxia‐inducible factor‐2 involved in chemo/radioresistance in solid tumors. Cancer Treat Rev. 2015;41:623‐633. doi:10.1016/j.ctrv.2015.05.004 25981453

[33] Dai H , Sheng X , Wang Y , et al. HIF1α regulates IL17 signaling pathway influencing sensitivity of Taxane‐based chemotherapy for breast cancer. Front Cell Dev Biol. 2021;9:729965. doi:10.3389/fcell.2021.729965 34595177 PMC8476907

[34] Tang Y , Tian W , Zheng S , et al. Dissection of FOXO1‐induced LYPLAL1‐DT impeding triple‐negative breast cancer progression via mediating hnRNPK/β‐catenin complex. Research (Wash D C). 2023;6:289. doi:10.34133/research.0289 PMC10726293 38111678

[35] Slack RJ , Macdonald SJF , Roper JA , Jenkins RG , Hatley RJD . Emerging therapeutic opportunities for integrin inhibitors. Nat Rev Drug Discov. 2022;21:60‐78. doi:10.1038/s41573-021-00284-4 34535788 PMC8446727

[36] Mitra SK , Schlaepfer DD . Integrin‐regulated FAK‐Src signaling in normal and cancer cells. Curr Opin Cell Biol. 2006;18:516‐523. doi:10.1016/j.ceb.2006.08.011 16919435

[37] Laursen LS , Chan CW , ffrench‐Constant, C . An integrin–contactin complex regulates CNS myelination by differential Fyn phosphorylation. J Neurosci. 2009;29:9174‐9185. doi:10.1523/JNEUROSCI.5942-08.2009 19625508 PMC4017644

[38] Wu H , Gu J , Zhou D , et al. LINC00160 mediated paclitaxel‐and doxorubicin‐resistance in breast cancer cells by regulating TFF3 via transcription factor C/EBPbeta. J Cell Mol Med. 2020;24:8589‐8602. doi:10.1111/jcmm.15487 32652877 PMC7412707

[39] Li W , Tanikawa T , Kryczek I , et al. Aerobic glycolysis controls myeloid‐derived suppressor cells and tumor immunity via a specific CEBPB isoform in triple‐negative breast cancer. Cell Metab. 2018;28:87‐103.e106. doi:10.1016/j.cmet.2018.04.022 29805099 PMC6238219

[40] Liu M , Li R , Wang M , et al. PGAM1 regulation of ASS1 contributes to the progression of breast cancer through the cAMP/AMPK/CEBPB pathway. Mol Oncol. 2022;16:2843‐2860. doi:10.1002/1878-0261.13259 35674458 PMC9348593

[41] Du R , Zhang X , Lu X , et al. PDPN positive CAFs contribute to HER2 positive breast cancer resistance to trastuzumab by inhibiting antibody‐dependent NK cell‐mediated cytotoxicity. Drug Resist Updat. 2023;68:100947. doi:10.1016/j.drup.2023.100947 36812747

[42] Xie J , Deng X , Xie Y , et al. Multi‐omics analysis of disulfidptosis regulators and therapeutic potential reveals glycogen synthase 1 as a disulfidptosis triggering target for triple‐negative breast cancer. MedComm (2020). 2024;5:e502. doi:10.1002/mco2.502 38420162 PMC10901283

[43] Gothlin Eremo A , Lagergren K , Othman L , Montgomery S , Andersson G , Tina E . Evaluation of SPP1/osteopontin expression as predictor of recurrence in tamoxifen treated breast cancer. Sci Rep. 2020;10:1451. doi:10.1038/s41598-020-58323-w 31996744 PMC6989629

[44] Zhang Y , Li S , Cui X , Wang Y . microRNA‐944 inhibits breast cancer cell proliferation and promotes cell apoptosis by reducing SPP1 through inactivating the PI3K/Akt pathway. Apoptosis. 2023;28:1546‐1563. doi:10.1007/s10495-023-01870-0 37486406

[45] Wei G , Tan M , Wang C , Liang L . Decreased miR‐127 promotes the occurrence of breast cancer via increasing the expression of SPP1. Adv Clin Exp Med. 2023;32:1113‐1123. doi:10.17219/acem/161161 36920269

[46] Chen C , Zhao S , Karnad A , Freeman JW . The biology and role of CD44 in cancer progression: therapeutic implications. J Hematol Oncol. 2018;11:64. doi:10.1186/s13045-018-0605-5 29747682 PMC5946470

[47] Nallasamy P , Nimmakayala RK , Karmakar S , et al. Pancreatic tumor microenvironment factor promotes cancer stemness via SPP1–CD44 Axis. Gastroenterology. 2021;161:1998‐2013.e1997. doi:10.1053/j.gastro.2021.08.023 34418441 PMC10069715

[48] Liu L , Zhang R , Deng J , et al. Construction of TME and identification of crosstalk between malignant cells and macrophages by SPP1 in hepatocellular carcinoma. Cancer Immunol Immunother. 2022;71:121‐136. doi:10.1007/s00262-021-02967-8 34028567 PMC10992184

[49] Zhang J , Liu F , Guo W , et al. Single‐cell transcriptome sequencing reveals aberrantly activated inter‐tumor cell signaling pathways in the development of clear cell renal cell carcinoma. J Transl Med. 2024;22:37. doi:10.1186/s12967-023-04818-9 38191424 PMC10775677

[50] Xie G , Yao Q , Liu Y , et al. IL‐6‐induced epithelial‐mesenchymal transition promotes the generation of breast cancer stem‐like cells analogous to mammosphere cultures. Int J Oncol. 2012;40:1171‐1179. doi:10.3892/ijo.2011.1275 22134360 PMC3584811

[51] Uchino M , Kojima H , Wada K , et al. Nuclear beta‐catenin and CD44 upregulation characterize invasive cell populations in non‐aggressive MCF‐7 breast cancer cells. BMC Cancer. 2010;10:414. doi:10.1186/1471-2407-10-414 20696077 PMC3087322

[52] Babaev VR , Ding L , Zhang Y , et al. Loss of 2 Akt (protein kinase B) isoforms in hematopoietic cells diminished monocyte and macrophage survival and reduces atherosclerosis in Ldl receptor‐null mice. Arterioscler Thromb Vasc Biol. 2019;39:156‐169. doi:10.1161/ATVBAHA.118.312206 30567482 PMC6344270

[53] Liu C , Zhang Y , Gao J , et al. A highly potent small‐molecule antagonist of exportin‐1 selectively eliminates CD44(+)CD24(−) enriched breast cancer stem‐like cells. Drug Resist Updat. 2023;66:100903. doi:10.1016/j.drup.2022.100903 36463808

[54] Elias D , Ditzel HJ . Fyn is an important molecule in cancer pathogenesis and drug resistance. Pharmacol Res. 2015;100:250‐254. doi:10.1016/j.phrs.2015.08.010 26305432

[55] Li X , Yang Y , Hu Y , et al. Alphavbeta6‐Fyn signaling promotes oral cancer progression. J Biol Chem. 2003;278:41646‐41653. doi:10.1074/jbc.M306274200 12917446

[56] Moon CS , Reglero C , Cortes JR , et al. FYN‐TRAF3IP2 induces NF‐κB signaling‐driven peripheral T cell lymphoma. Nat Can. 2021;2:98‐113. doi:10.1038/s43018-020-00161-w PMC8081346 33928261

[57] Wang Z , Wu Z , Wang H , et al. An immune cell atlas reveals the dynamics of human macrophage specification during prenatal development. Cell. 2023;186:4454‐4471.e4419. doi:10.1016/j.cell.2023.08.019 37703875

[58] Hughes R , Qian BZ , Rowan C , et al. Perivascular M2 macrophages stimulate tumor relapse after chemotherapy. Cancer Res. 2015;75:3479‐3491. doi:10.1158/0008-5472.Can-14-3587 26269531 PMC5024531

[59] DeNardo DG , Brennan DJ , Rexhepaj E , et al. Leukocyte complexity predicts breast cancer survival and functionally regulates response to chemotherapy. Cancer Discov. 2011;1:54‐67. doi:10.1158/2159-8274.Cd-10-0028 22039576 PMC3203524

