# Supplementary figures and images for: Single‐cell transcriptome sequencing reveals SPP1‐CD44‐mediated macrophage–tumor cell interactions drive chemoresistance in TNBC
Source: J Cell Mol Med. 2024 Jul 9;28(13):e18525. doi: 10.1111/jcmm.18525 (PMC11233263; doi:10.1111/jcmm.18525)

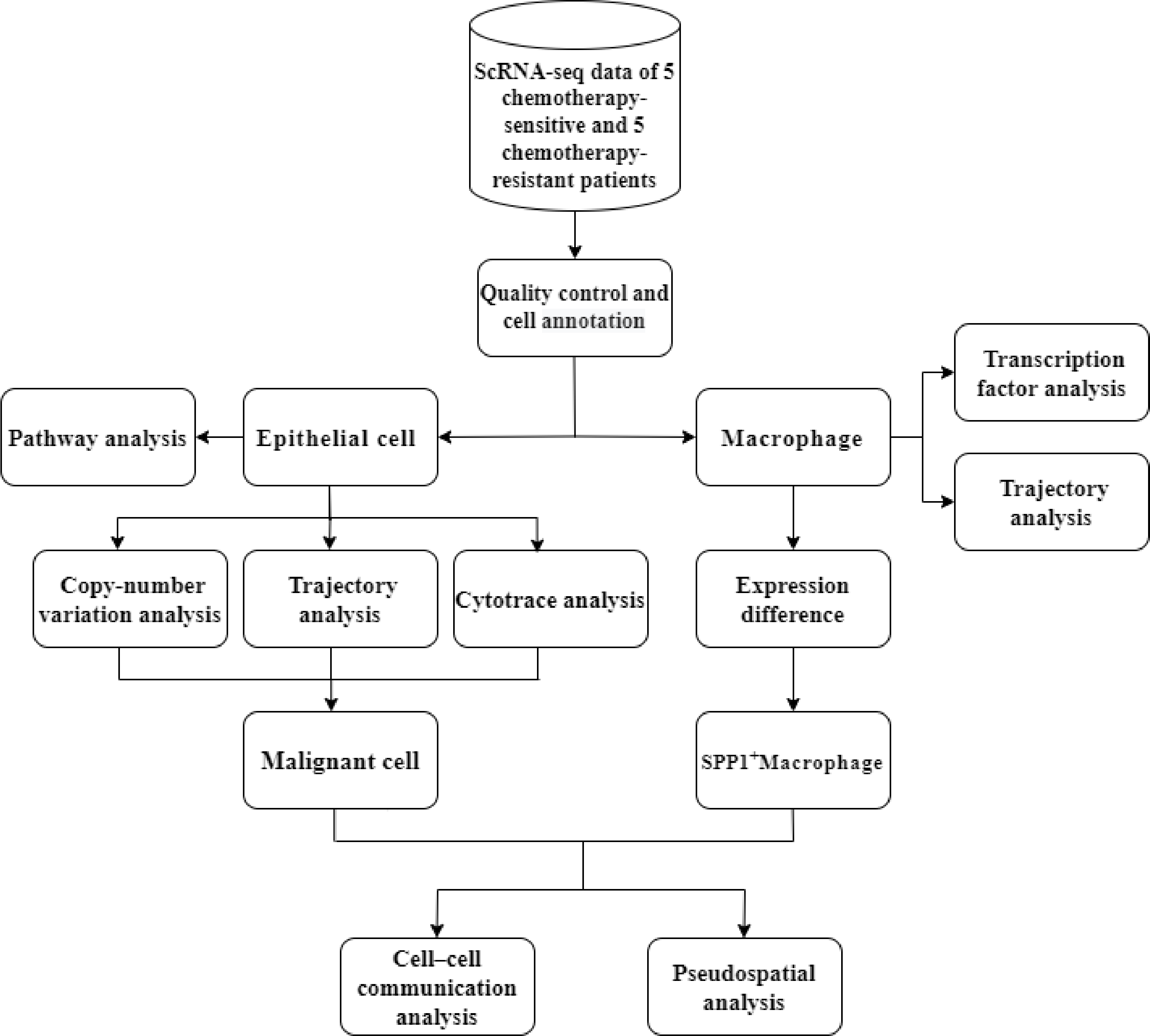

Supplement: Supplementary file 1 — Figure S1. [file JCMM-28-e18525-s003.tif]

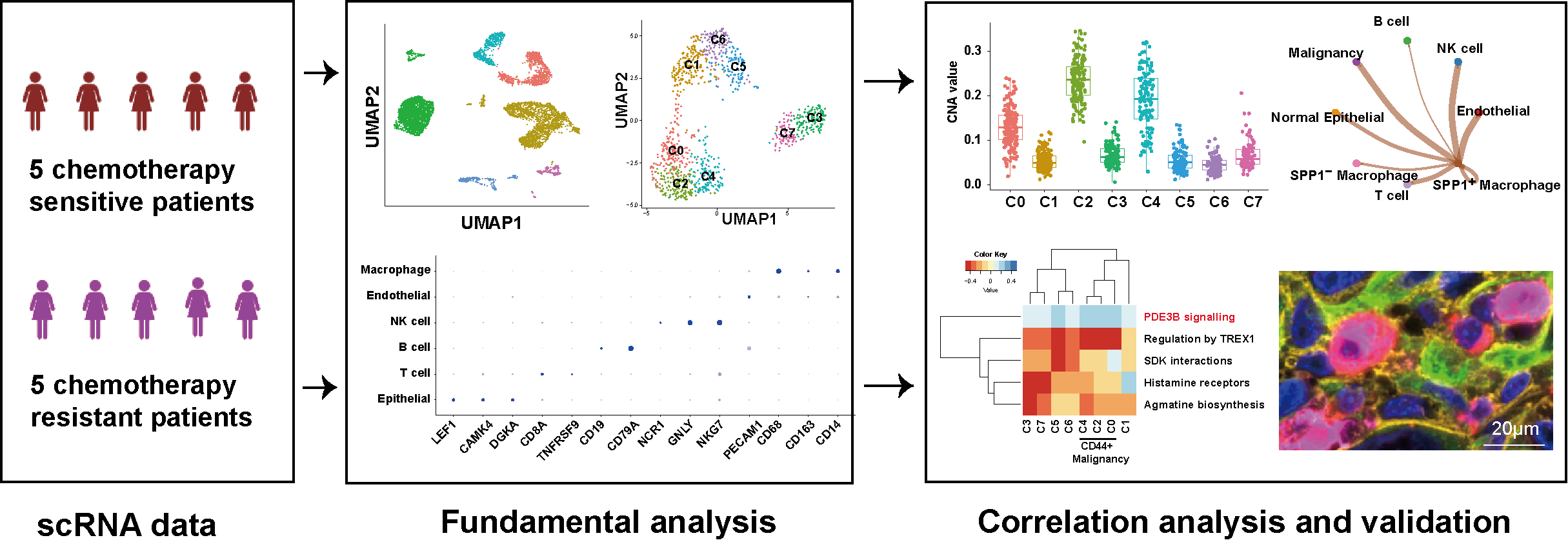

Supplement: Supplementary file 2 — Figure S2. [file JCMM-28-e18525-s004.tif]
